# Persistent Postpartum Urinary Retention: A Case Report and Review of Literature

**DOI:** 10.7759/cureus.57956

**Published:** 2024-04-10

**Authors:** Dimitris Baroutis, Rafail Mantzioros, Michael Sindos, Alexandros Psarris, George Daskalakis

**Affiliations:** 1 1st Department of Obstetrics and Gynecology, Alexandra Hospital, National and Kapodistrian University of Athens, Athens, GRC

**Keywords:** epidural anesthesia, pudendal nerve, intermittent catheterization, voiding dysfunction, postpartum urinary retention

## Abstract

This case report describes persistent urinary retention lasting over 30 days postpartum in a 23-year-old primiparous female after an otherwise uncomplicated vaginal delivery at 37 weeks gestation. Notable risk factors present included epidural anesthesia, episiotomy, third-degree perineal laceration, and inability to void spontaneously before leaving the delivery room. Despite initial catheterization draining a large volume, the patient experienced recurrent failed voiding trials requiring ongoing intermittent catheterization during her admission. One month after delivery, voiding trials were finally successful, and she regained normal spontaneous voiding without catheterization. This case highlights persistent postpartum urinary retention (PUR) as an uncommon but potentially serious obstetric complication requiring prompt diagnosis and appropriate management to prevent adverse events and optimize outcomes. Although most cases are self-limited, a high index of suspicion is needed to institute timely treatment with intermittent catheterization given the morbidity associated with sustained bladder overdistension postpartum.

## Introduction

Postpartum urinary retention (PUR) is a concerning complication that can occur after childbirth, yet there is a lack of consensus on the precise definition and diagnostic criteria for this condition. While some authors define PUR as the inability to void spontaneously within six hours of delivery or after catheter removal, referred to as overt PUR, others argue that increased post-void residual urine volume (PVR) after spontaneous micturition, known as covert PUR, should also be considered as evidence of impaired bladder emptying [1-3]. The appropriate cutoff for abnormal PVR is controversial, with emerging evidence suggesting 500 mL rather than the traditional 150 mL may be a more meaningful indicator of impaired voiding [2,3]. Reported incidence rates for PUR vary widely from 0.05% to 45%, likely reflecting this discordance in definitions [4]. In some cases, PUR persists beyond the initial postpartum period, with retention continuing for days or even weeks after delivery [5]. The underlying pathophysiology of PUR involves a complex interplay of anatomical, neurological, and hormonal factors that can impair detrusor contractility and urinary sensation in the postpartum period. Resultant bladder overdistension and large PVR volumes further inhibit spontaneous voiding through maladaptive neural reflexes. If unresolved, PUR can lead to serious complications including urinary tract infection, bladder damage, and renal impairment [6]. Despite the significant morbidity associated with PUR, this condition often goes unrecognized and undertreated due to the lack of consensus on diagnostic criteria and management protocols.

In this report, we present a case of persistent PUR continuing weeks after delivery and review the current literature regarding the definition, diagnosis, incidence, predisposing factors, and management of PUR. This case highlights the importance of increased awareness and evidence-based recommendations for this frequent yet poorly understood postpartum complication. A thorough assessment of voiding function and early intervention when impaired bladder emptying is detected are crucial to prevent short- and long-term sequelae of sustained PUR.

## Case presentation

A 23-year-old primiparous female at 37+2 weeks gestation presented with contractions. Her history included a cervical cerclage at 19 weeks for shortening and sickle cell trait. As the intensity of contractions increased, she was transferred to labor and delivery, the cerclage was removed, and a microbiological culture was obtained. Early epidural anesthesia was administered at 3 cm dilation using ropivacaine/fentanyl at L3-L4. Four hours later, she delivered a healthy 2,880 g boy spontaneously, with median episiotomy, due to head expulsion difficulty, and 3a perineal laceration repair. The epidural was discontinued 30 minutes after delivery.

Catheter removal on day 1 was followed by reinsertion for failed spontaneous voiding despite mobilization and analgesia. Testing was normal. The catheter was removed again after 24 hours. The patient initially reported voiding then presented with oliguria and pain. Abdominal examination revealed a palpable mass in front of the uterus. Immediate catheterization drained 450 mL and gradually 2,000 mL in total, relieving her pain. Recurrent retention followed repeated removal attempts, prompting neurological examination and lower spine magnetic resonance imaging (MRI) showing no abnormalities. Vitamin B12 and folate were normal. After a urogynecology consultation, she was discharged with an indwelling catheter, and a urodynamic follow-up was scheduled.

She returned on day 23 postpartum with suprapubic pain and a positive urine culture. Antibiotics were initiated per sensitivity results after the catheter was changed. After normal urodynamics and minimal residual volume, the catheter was removed on day 34, and she was discharged without medications. One month post-discharge, she remained asymptomatic, suggesting resolving postpartum urinary retention.

The timeline of events is shown in Table 1.

**Table 1:** Timeline of key events in the clinical course

Postpartum day	Events
Delivery	37+2 weeks gestation, spontaneous vaginal delivery with epidural anesthesia, median episiotomy, third-degree perineal laceration repair
Day 1	Failed spontaneous voiding, insertion of transurethral catheter
Day 2	Removal of catheter, failed voiding trial, reinsertion of transurethral catheter
Day 3	Catheter removal, patient reported voiding, developed oliguria, abdominal pain and palpable bladder, insertion of transurethral catheter, drained 2,000 mL of urine
Day 4-33	Multiple failed voiding trials after catheter removal, indwelling transurethral catheterization
Day 33	Suprapubic pain and catheter change, positive urine culture, treatment with antibiotics per sensitivity, catheter changed
Day 34	Normal urodynamic testing, minimal post-void residual, catheter removed, discharged home
Day 58	1 month follow-up, normal voiding restored

## Discussion

Persistent postpartum urinary retention (PUR) is a rare occurrence, with reported incidence ranging from 0.05% to 0.18% [5,6]. Timely diagnosis and management are crucial to prevent complications such as detrusor muscle and nerve damage, bladder rupture, and kidney failure [2-4,7].

The proposed pathogenesis of PUR includes pudendal nerve injury during childbirth, leading to prolonged terminal motor latencies persisting for months postpartum [8-10]. Recent data not only confirm these factors but also highlight additional contributors to PUR, including the absence of spontaneous voiding before leaving the delivery room, vulvar edema or perineal hematoma, birth weight > 3.5 kg, a longer duration of the second stage of labor, and second-degree or greater perineal laceration [2-4,11]. Specifically, epidural analgesia, prolonged second stage of labor, and instrumental delivery appear notably associated with persistent postpartum urinary retention [5,6].

In our case, various predisposing risk factors were identified. The patient, a primigravida, received epidural analgesia, underwent an episiotomy, and suffered a third-degree perineal tear. Unfortunately, she was unable to void spontaneously before leaving the delivery room, and as a consequence, the urinary catheter was not removed until the next day. Attempts were made to encourage voiding, including the use of oral analgesics, mobilization, and patient privacy, but without success. Afterward, a transurethral catheter was inserted and left in place for 24 hours; however, the patient declined the recommended approach of clean intermittent self-catheterization (CISC), as proposed due to its shorter median duration of catheterization compared to the indwelling one [12,13].

Our patient's predisposing factors align with established risk factors for persistent postpartum urinary retention, as outlined in Table 2.

**Table 2:** Risk factors for persistent postpartum urinary retention

Risk factors from the literature	Patient's characteristics
Primiparity [1]	Primiparous
Epidural analgesia [1,5,6]	Received epidural analgesia
Instrumental delivery [1,5,6]	Spontaneous vaginal delivery
Episiotomy [1]	Received episiotomy
Failed voiding before leaving the delivery room [2,4]	Unable to void before leaving the delivery room
Birth weight > 3.5 kg [2,4]	Birth weight = 2.88 kg
Prolonged second stage [2,4]	Duration of the second stage = 30 minutes approximately
Second-degree or greater laceration [2,4,11]	Third-degree perineal laceration

Rarely, long-term dysfunction occurs, involving mechanical obstruction from hematoma/edema or bladder descent, as well as functional impairment from birth-related pelvic floor pain, urethral overactivity, and reduced bladder filling awareness. Detrusor underactivity from nerve damage or neglected overdistension may also occur, along with atony from increased postpartum progesterone [3,4,6].

The approach to treating evident PUR involves intermittent catheterization, as pharmacological therapies are ineffective. Catheterization is recommended for a palpable bladder and difficulty voiding or oliguria. There is no established protocol; four to six hourly catheterization or as needed when the urge to void occurs is typical. With small spontaneous volumes, self-catheterization to assess residual volume is instructed. Discontinuation is considered when the residual volume is <150 mL and significant voiding difficulty resolves, based on empirical observations lacking evidence-based standards [14,15]. Routine antibiotic prophylaxis is deemed unnecessary [16].

PUR typically follows a self-limiting course, resolving in about one week [14,17]. In covert PUR, 96%-100% normalize residual volumes within 2-5 days [14,18].

## Conclusions

In summary, this case illustrates persistent postpartum urinary retention as an uncommon but serious complication after vaginal delivery. Risk factors such as primiparity, epidural use, episiotomy, and perineal lacerations likely contributed. Prompt diagnosis and intermittent catheterization prevented adverse events. The self-limited natural history involves resolution within 1-2 weeks postpartum, likely due to transient pudendal nerve impairment improving detrusor contractility. Increased awareness of persistent PUR is important to minimize morbidity through timely treatment. Avoiding unnecessary interventions such as episiotomy and judicious epidural use may reduce incidence. Excellent outcomes can be achieved with proper diagnosis and management.
